# Genitourinary Diseases and Syphilis

**Published:** 1902-08

**Authors:** 


### BOOK REVIEWS.

Genitourinary Diseases and Syphilis. For students and practitioners. By Henry H. Morton, M. D., Clinical professor of Genitourinary Diseases in the Long Island College Hospital; Genitourinary Surgeon to the Long Island College and Kings County Hospitals and the Polhemus Memorial Clinic. Illustrated with half-tones and full-page color plates. Pages xii—372. Philadelphia: F. A. Davis Co., 1902. [Price, $3.00 net, delivered.]

In this volume the author has taken up and considered the subjects in a way that leads one to think that he has had considerable clinical experience and been a close observer; yet, nevertheless, he must be differed from upon a few points that although they may be considered trivial are very important as regards the patient's welfare. In the treatment of chronic specific urethritis the author still clings to the use of the catheter. It is known that a catheter always produces irritation; why not then dispense with it when the same results are obtained without it? As silver nitrate always acts as an irritant for the first few hours, even in dilute solutions, Morton's readers would reach better and quicker results if he had advised commencing the treatment of either simple or chronic specific urethritis with a 1-15000, instead of a 1-4000 solution.

Mention should have been made of those very important little agents, leeches, in the treatment of acute prostatitis, for with these resolution is brought about in a few hours instead of taking days as under the advised method. Such an important subject as chronic prostatitis should have been considered more fully and possibly more clearly.

The chapter on stricture is well written and careful observers will readily agree that the advice that it is always best to dilate, when results can be obtained, rather than to cut, is sound.

Morton, we think, should have gone more deeply into the subject of diet and constitutional habits as predisposing factors in the production of stone, especially the oxalic and uratic varieties. There is no doubt that malassimilation is accountable for a goodly number of all urinary calculi of the forms mentioned. Only two lines are given to the consideration of the urine as a diagnostic agent in the detection of stone. It strikes the reviewer that instead of dilating upon diagnostic symptoms well known and published for years, that methods which are comparatively new, yet of most positive value, should have received more attention than the author has given them.

Inflammatory diseases of the bladder are carefully considered, yet a few recent findings, and now acknowledged to be almost pathognomonic, medically and symptomatically, have not been mentioned.

In speaking of pyelitis, Morton seems to have lost sight of the fact that involvement of the kidney pelvis often develops suddenly, for instance during an acute attack of gonorrhea. In this connection the author says: "Pain is rarely a prominent symptom unless stone be present,'' whereas it is in reality the most important symptom, is very severe, is located in the affected side and directly over the kidney. Clumping of the pus cells should have been referred to as seen with the microscope in this condition, for unless the urine is very opaque this one point is far more important than any information that can be obtained from the cystoscope.

Considerable space is given to syphilis, the subject being well delineated and in a manner that shows careful study of many cases.

The author closes the book with a chapter on sexual diseases. These subjects are not considered exhaustively yet they give a clear working basis for the general practitioner or student. On the whole, the work may be considered practically up to date and in almost every part it gives the reader a good understanding of the diagnosis and treatment of disease of the genitourinary organs. J. H. D.

